# Low-density asymptomatic parasitemia in southern Zambia does not lead to clinical malaria and is not associated with household transmission: results from a two-year cohort study

**DOI:** 10.21203/rs.3.rs-8641961/v1

**Published:** 2026-01-30

**Authors:** Jessica L. Schue, Anne C. Martin, Japhet Matoba, Caison Sing’anga, Mukuma Lubinda, Ben Katowa, Michael Musonda, Sophie Berube, Timothy Shields, Tamaki Kobayashi, Harry Hamapumbu, Edgar Simulundu, Douglas E. Norris, Amy Wesolowksi, William J. Moss

**Affiliations:** Johns Hopkins Bloomberg School of Public Health; Johns Hopkins Bloomberg School of Public Health; Macha Research Trust; Macha Research Trust; Macha Research Trust; Macha Research Trust; Macha Research Trust; University of Florida; Johns Hopkins Bloomberg School of Public Health; Johns Hopkins Bloomberg School of Public Health; Macha Research Trust; Zambia National Public Health Institute; Johns Hopkins Bloomberg School of Public Health; Johns Hopkins Bloomberg School of Public Health; Johns Hopkins Bloomberg School of Public Health

**Keywords:** malaria, asymptomatic, subpatent, elimination

## Abstract

**Background:**

In low malaria transmission settings targeting elimination, the World Health Organization recommends a combination of mass (e.g., mass test-and-treat), targeted (e.g., chemoprophylaxis or treatment for travelers), and reactive (e.g., reactive drug administration) strategies. Most of these strategies would not identify and treat individuals with asymptomatic parasitemia. This study was conducted in a pre-elimination setting in Southern Province, Zambia to examine risk factors for asymptomatic parasitemia, its epidemiologic relationship to incident clinical malaria, and evidence of its contribution to ongoing transmission to inform policy on whether these parasitemic individuals need to be identified and treated to achieve malaria elimination.

**Methods:**

An intensive longitudinal cohort study of 197 households within the catchment area of a single health center was designed to capture all clinical malaria cases and episodes of asymptomatic parasitemia between 2018 and 2020. During monthly collections, all household members and overnight visitors were administered a questionnaire and a blood sample was collected to identify *Plasmodium falciparum* parasitemia by qPCR. Passive surveillance was also established at the local health center to identify cases of clinical malaria. The statistical analysis examined risk factors for parasitemia and associations between asymptomatic parasitemia and subsequent episodes of clinical malaria within individuals and parasitemia in household members.

**Results:**

Of the 1071 individuals enrolled in the cohort, 144 (13%) were positive by qPCR for *P. falciparum* at least once during the two-year study period. Monthly parasite prevalence by qPCR never exceeded 4% and parasite density was very low with a median of four parasites/μL. Incidence of self-reported clinical malaria was 46.7 cases per 1000 person-years. Low-density asymptomatic parasitemia was identified in all age groups, including young children. There was no association between asymptomatic parasitemia and clinical malaria within individuals, nor was there an association between asymptomatic parasitemia and subsequent parasitemia in household members beyond one month of the index case.

**Conclusion:**

Low-level parasitemia was prevalent despite few cases of clinical malaria in this low transmission setting. There was no evidence that low-level asymptomatic parasitemia led to clinical cases of malaria or transmission to other household members.

## Background

As of 2025, 47 countries achieved malaria elimination through a combination of mass, targeted, and reactive interventions (1). Understanding the causes of residual transmission in an area approaching elimination is necessary to deliver appropriate interventions and ultimately achieve malaria elimination. Residual transmission may be due to repeated parasite introductions via imported cases or ongoing low-level transmission from symptomatic infections prior to their treatment and/or untreated chronic, asymptomatic infections (2). These drivers are countered most effectively with different interventions. For example, reactive test-and-treat can curb transmission introduced from imported cases and symptomatic infections, whereas mass drug administration (MDA) can clear infections that may not result in clinical symptoms or be detected by available diagnostics and thus act as reservoirs for transmission (3–5).

Asymptomatic infections with moderate levels of parasitemia are major gametocyte reservoirs in high and moderate transmission settings and contribute disproportionately to onward transmission (6–8). In low transmission settings, where individuals have less protective immunity against clinical disease, asymptomatic parasitemias are typically low-density (9), undetectable by point-of-care testing (10), and linked to persistent carriage across transmission seasons (11, 12), but are of unknown clinical and public health relevance. Asymptomatic parasitemia in low transmission settings could be important if they are a prelude to clinical malaria in the infected individual or a source of transmission to other individuals. However, few cohort studies have been conducted in pre-elimination settings to understand the relationships between asymptomatic parasitemia and subsequent clinical malaria or asymptomatic parasitemia and subsequent parasitemia in other household members.

This work describes a prospective longitudinal cohort study designed to capture all cases of clinical malaria and asymptomatic subpatent parasitemia in a geographically defined population in Southern Province, Zambia. Background prevalence of asymptomatic parasitemia with *Plasmodium falciparum* is 1–3% by quantitative PCR (qPCR) (13). Specifically, this study seeks to guide elimination strategies in low transmission settings through examination of asymptomatic parasitemia: its risk factors, its epidemiologic relationship to incident clinical malaria, and evidence of its linkage to ongoing transmission.

## Methods

### Study Site

**Figure 1:**
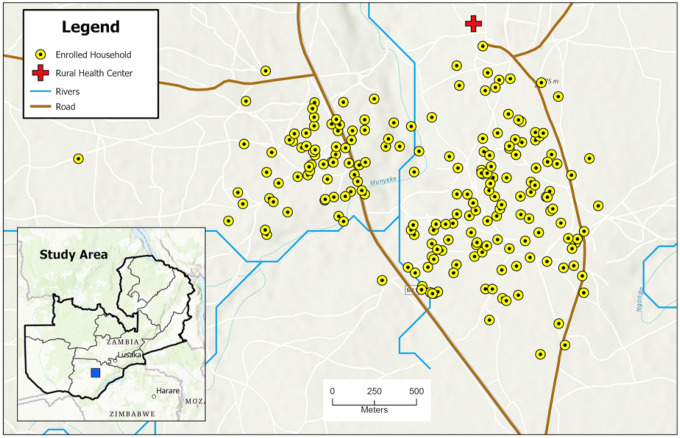
Map of enrolled households in the catchment area of Mapanza Rural Health Center.

A 24-month longitudinal cohort study was conducted from October 2018 through September 2020 in Choma District, Southern Province, Zambia, a low-transmission setting typical of southern Zambia. The region has a tropical savannah climate with a rainy season from December to April, followed by a cool dry season from May to August, and a hot dry season from September to November. The primary malaria vector is *Anopheles arabiensis*, which peaks during the rainy season (14, 15). The study site was a contiguous two-square-kilometer area, defined by natural borders of roads and footpaths, located within the catchment of Mapanza Rural Health Centre (RHC) (Fig. 1).

### Household and participant selection

All households within the study site were enumerated using satellite imagery and were invited to participate. Geocoordinates of consenting households were captured by global positioning system (GPS) devices. Throughout the study period, newly constructed and newly occupied households in the study area also were invited to participate. Enrolled households were visited monthly for a minimum of one year. If a household agreed, the study visits continued into a second year.

All household residents and overnight visitors older than three months of age were invited to participate. Participants were classified as either a permanent resident, temporary resident (household resident for 2 months or more), or visitor (household resident for fewer than 2 months). Individuals younger than three months or with severe illness other than malaria were excluded. Written informed consent was obtained from each participant 16 years of age or older. Parental or guardian permission was obtained for all children younger than 16 years of age, and assent was obtained from children 13 to 15 years old.

### Cohort data collection

Household and individual surveys were administered monthly, except between April and July 2020 when community data collection was paused in compliance with the Government of the Republic of Zambia’s COVID-19 prevention measures. The household survey recorded household size, amenities, net ownership, and history of indoor residual spraying. Each visit captured the number and condition of nets in the household, where household members slept, and any visitors to the household. House construction variables such as roof and wall material were collected for each sleeping structure within a household complex. Individual surveys captured demographic characteristics, socio-economic indicators, use of nets, times spent indoors versus outdoors, travel history, recent illnesses, and health care seeking behavior. Travel history included the location, purpose, and duration of up to four trips during the previous month. Each participant also had their tympanic temperature taken, and those with a temperature at 38° Celsius or higher were administered a malaria rapid diagnostic test (RDT) (SD Bioline Malaria AG *P.f*., Abbott, Abbott Park, Illinois, USA). Participants who tested positive were offered treatment with artemether-lumefantrine (16). Pregnant women and children under 5 kilograms who tested positive were to be offered transportation to the health center treatment. Dried blood spots (DBS) were collected monthly for detection of *P. falciparum* parasitemia by qPCR.

### Health center symptomatic surveillance

Passive case detection was established at Mapanza RHC, the catchment area of which included the cohort households, and its aflliated health posts. Individuals older than three months of age who tested positive for malaria by RDT were asked to participate in the study by the health center staff. Those who provided written consent were administered a brief questionnaire, including basic demographic information, recent travel, bed net use, recent illnesses, and whether or not they were participants in the cohort study. DBS were collected from all consenting patients for the detection of *P. falciparum* by qPCR.

### Laboratory Testing

DNA was extracted from the DBS using a standard saponin and Chelex-100 extraction procedure, and qPCR targeting the *P. falciparum cytochrome-b* gene was done using SYBR Green PCR Master Mix (Applied Biosystems, Thermo Fischer Scientific Inc, Waltham, MA) (10). All samples were run in duplicate. Parasite density was estimated based on the cycle threshold, and samples were considered positive if at least one of the two wells had a parasite density above one parasite/μL and a melting point within ± 0.5° Celsius of the melting point of the 3D7 g-DNA control. Positive samples were run on gel electrophoresis to confirm the DNA product size.

### Data Analysis

Parasite prevalence by qPCR and the incidence of clinical malaria using health center surveillance were calculated across the study period. Monthly parasite prevalence (the proportion of participants parasitemic in a given month) and period prevalence (the proportion of participants ever-parasitemic) were calculated. Annual incidence of clinical malaria was calculated using the cohort cases captured in health facility surveillance and by self-reported confirmed malaria diagnoses at locations other than Mapanza RHC. These were summed and divided by the person-months of the study period (excluding the months the study was paused) and multiplied by twelve to estimate the annual incidence.

Descriptive and statistical analysis evaluated risk factors for asymptomatic parasitemia and evidence of the clinical and public health relevance of asymptomatic parasitemia as a prelude to clinical malaria or a source of transmission to other household members. To explore risk factors for asymptomatic parasitemia, multiple analyses were performed. First, summary statistics were stratified by participants who were ever versus never positive by qPCR to examine univariate risk factors associated with being ever positive. Differences were compared using Fisher’s exact test for proportions and the Kruskal-Wallis test for continuous data. Second, univariate regression was used to test for associations between qPCR positivity and age group, travel, and net usage. Finally, to assess if parasitemia occurred randomly across the population, a random forest model was constructed, maximizing predictive power for qPCR using available covariates, and the model fit was compared between the true study population (covariates and associated qPCR outcomes) and an alternate population where the covariates and associated qPCR outcomes were unlinked and qPCR outcomes were randomly assigned.

The possible clinical relevance of asymptomatic parasitemia as an indicator of subsequent or prior clinical malaria in the parasitemic individual was explored by examining the association between qPCR positivity and two individual-level outcomes in the six months surrounding the positive event: symptoms reported and self-reported malaria diagnosis. Logistic regression was used to examine the odds ratio of each outcome in those parasitemic by qPCR compared to those who were qPCR negative in each of the three months preceding and following the month in which the outcome was measured. All analyses adjusted p-values for multiple comparisons using a Bonferroni approach.

To explore the possible public health importance of asymptomatic parasitemia as a source of transmission to other individuals, associations were examined between individual qPCR positivity and three outcomes in other household members during the three months *following* the positive event, including symptoms reported, self-reported malaria diagnosis, and qPCR positivity, using the same statistical approach as described above for associations with clinical malaria.

To account for potential false positive qPCR results, a sensitivity analysis was conducted for which qPCR positivity was defined as any qPCR positive test with a parasite density of at least 10 parasites/μL. All analyses were done using R version 4.2.3.

## Results

A total of 201 households, some comprised of multiple structures, were identified during the two years of the study, 197 were enrolled, and complete monthly data were obtained for 167 (83%) households. Over the 24 months of the study, 1198 persons were screened and 1071 (89%) were enrolled. Median participant age was 16 years (IQR: 8–28), and 58% of participants were female (Table 1). Median household size was 5 people (IQR: 3–7, range: 1–18).

### Incidence of clinical malaria was low

The incidence of clinical malaria in the cohort was 46.7 cases per 1000 person-years, with an incidence of 24.9, 27.1, and 50.7 cases per 1000 person-years in individuals younger than 5 years old, 6 to 15 years, and 15 years and older, respectively. Mapanza RHC recorded 206 confirmed cases of malaria by RDT, of which only four were participants in the cohort study. These four cases occurred during the COVID-19 pandemic when data collection was paused (Table S1). An additional forty cohort members reported a malaria diagnosis at a different health care facility, most commonly, nearby Macha Hospital. None of these forty were qPCR positive in the month of, the month preceding, or the month following their self-reported diagnosis of malaria, although most (n = 34) reported taking Coartem^®^ (artemisinin-lumefantrine), which rapidly clears parasitemia. Three reported finishing their course of medication the day prior to the study visit, ten reported having finished their treatment course within the prior week, and 21 completed their treatment more than one week prior to the study visit. An additional four adult cohort participants reported taking antimalarial medications from a traditional healer, friend, family member, or local chemist and were not included in the case count.

### Parasite prevalence and density were low in those with asymptomatic parasitemia

**Figure 2:**
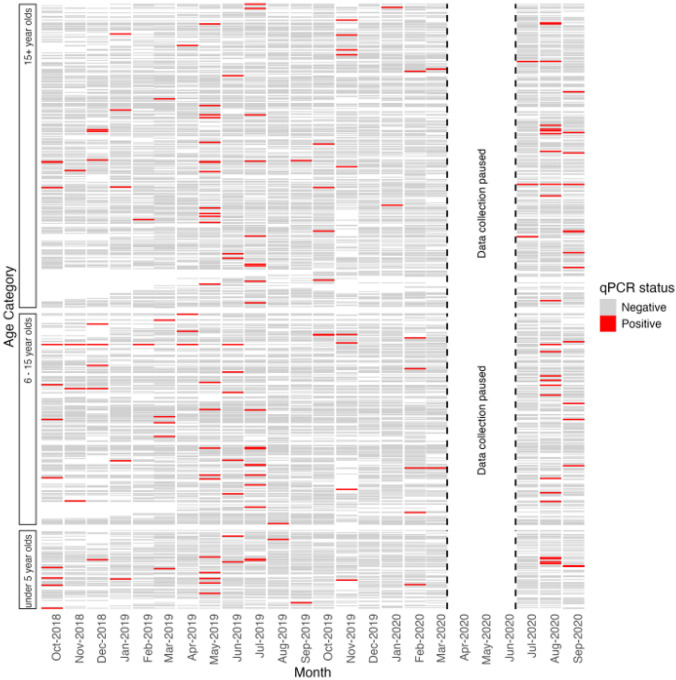
Individual qPCR results of cohort members over time for those who were ever positive. Each line represents one individual cohort participant ordered by age. Cohort data collection was paused from April to July in 2020 in compliance with the Government of Zambia’s COVID-19 prevention measures.

**Figure 3:**
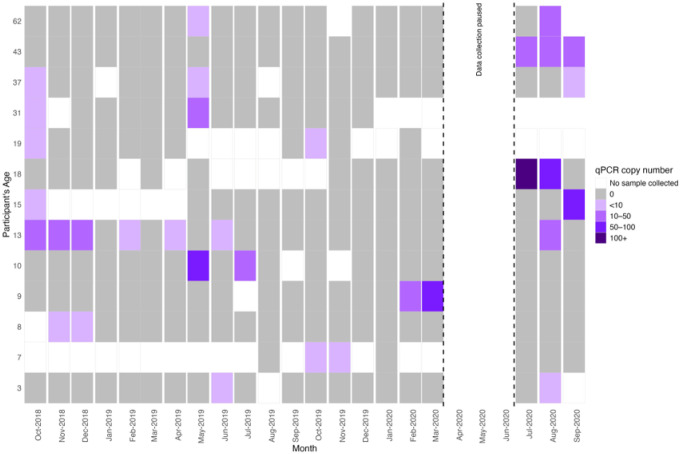
Frequency and timing of parasitemia by qPCR among individuals who tested positive more than once. None self-reported or had confirmed clinical malaria.

There were 164 episodes of parasitemia identified by qPCR in 144 individuals (Table 1, 13.3% of individuals). No RDT positive participants were identified in the cohort despite testing those who were febrile at the time of the study visit (n = 43). Monthly mean parasite prevalence was 1.5% and highest in May 2019 (4.2%), July 2019 (3.5%), and August 2020 (4.4%) (**Figure S1**). Most (89%) individuals who were ever parasitemic were only positive once (Figure 2). Fifteen individuals were parasitemic on more than one study visit, for an average of 2.4 monthly visits (Range: 2 – 7) (Figure 3), although none of these individuals self-reported having clinical malaria during the study period.

**Figure 4:**
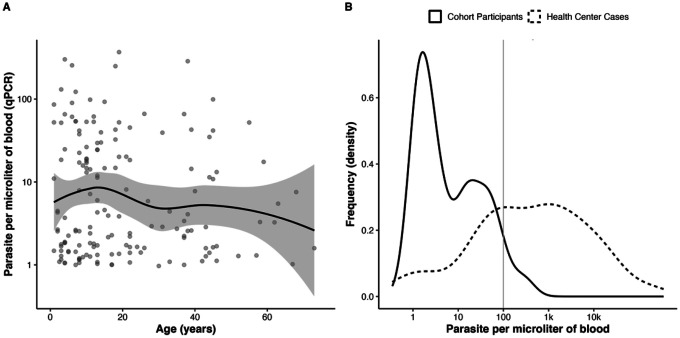
Parasitemia levels by age among qPCR positive cohort members A) Parasitemia by qPCR in log base 10 by age with a Loess smoothing line for trend. B) Raw parasite density frequency plots stratified by cohort participants and health center cases. The blue line represents cohort participants, and the red line represents health facility cases. The dotted line is 100 parasites/μL, the accepted limit of detection of RDTs.

The median parasite density was only four parasites/μL (IQR: 2 – 23) (Figure 4, **Panel B**), much lower than the parasite density for clinical cases at Mapanza RHC (393 parasites/μL, IQR: 41 – 3340). Most (96%) qPCR positive individuals had parasitemia levels below 100 parasites/μL. There was a decreasing trend but no statistical correlation between age and level of parasitemia (Figure 4, **Panel A**) (Pearson correlation coeflcient, r = −0.13).

### While travel, household visitors, and bed net use varied, none were risk factors for parasitemia

The odds of parasitemia by qPCR were not different among those who reported travel (odds ratio: 0.82, p-value= 0.45), nor among individuals living in households with others who had travelled (odds ratio: 1.0, p-value= 0.77). The odds of parasitemia by qPCR were not higher in visitors (odds ratio: 1.3, p-value= 0.59) nor in individuals living in households that had a visitor (odds ratio: 1.2, p-value= 0.46). Using a net was not associated with parasitemia by qPCR (odds ratio: 1.0, p-value= 0.34).

### Common symptoms of malaria were prevalent but not associated with parasitemia

**Figure 5:**
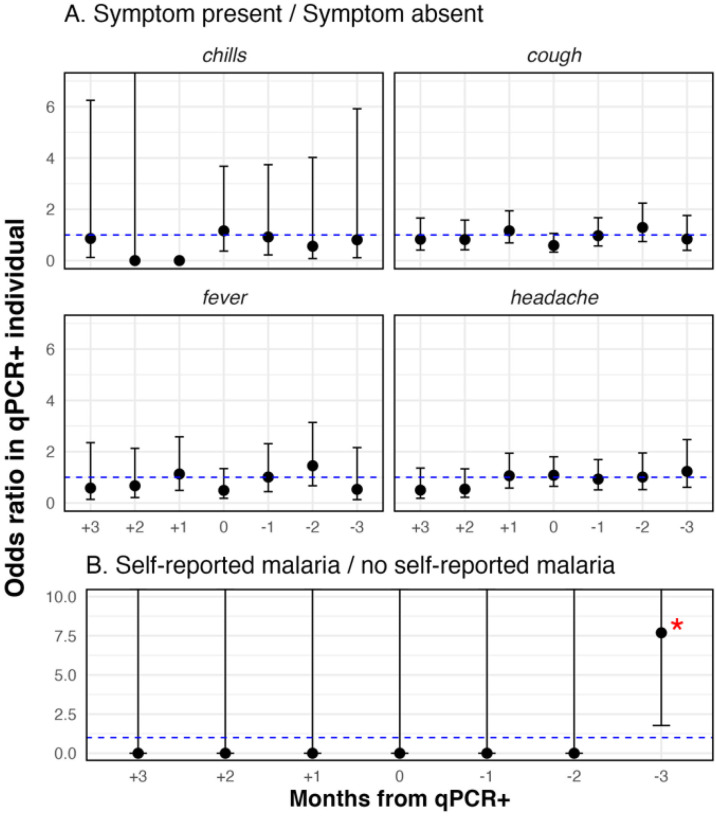
No associations between individual qPCR positivity and clinical disease

There was no statistical association with current or lagged parasitemia and fever, cough or headache (Figure 5, **Panel A**). The prevalence of fever was 7.5% in children younger than 5 years, 3.2% in children 5–15 years, and 5.1% in those older than 15 years. There were 43 instances of fever at the time of the study visit among 40 household residents, but only one febrile individual was parasitemic by qPCR.

Univariate analysis examined associations between parasitemia by qPCR and clinical outcomes to assess evidence of the clinical relevance of qPCR positivity. The alpha for the significance threshold was adjusted for multiple comparisons. A) qPCR positivity and symptoms of malaria B) qPCR positivity and self-reported malaria. Participants were asked to self-report malaria diagnoses received at a health facility.

### There was no evidence that those with parasitemia progressed to clinical malaria

Most (97%) of the 147 individuals ever parasitemic by qPCR did not report any episodes of clinical malaria during the 24-month study period. Specifically, no qPCR infected individuals reported clinical malaria in the three months prior or two months following their episode of parasitemia. Three months after individuals self-reported a diagnosis of clinical malaria, their odds of having parasitemia by qPCR were higher than the odds of parasitemia by qPCR in individuals with no history of clinical malaria (univariate odds 7.6, CI [1.8, 33.5] p-value = 0.0066) (Figure 5, **Panel B**), but there was no association in the months preceding the diagnosis of clinical malaria nor during the first or second month following diagnosis.

### There was no evidence that parasitemia was associated with subsequent clinical malaria in other household members

**Figure 6:**
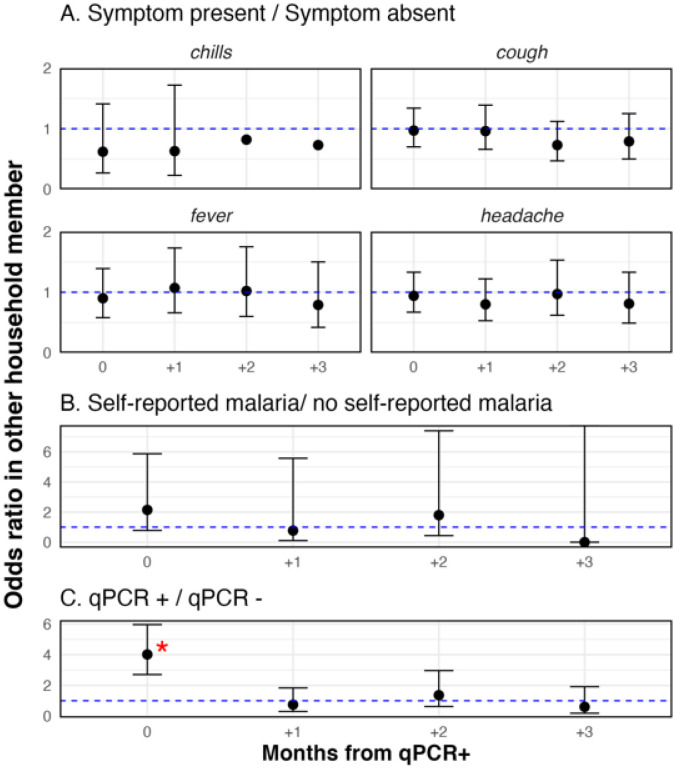
No associations between individual parasitemia by qPCR and clinical malaria or subsequent parasitemia in household members.

There was no statistical association between parasitemia by qPCR and symptoms of malaria or self-reported malaria in other household members in the three months following the qPCR positive test result (Figure 6, Panels A, B). Individuals with parasitemia by qPCR clustered at the household level, where in a households with a qPCR positive individual, the odds of having other positive members in the same month were four times the odds of having no positive household members that month (Odds ratio: 4.0, 95% CI: 2.7, 6.0). However, there were no higher odds of household members being parasitemic by qPCR in the three months following parasitemia in the index case, suggesting no association with household transmission.

Univariate analyses examined associations between qPCR positivity and outcomes in other household members to examine whether parasitemia led to onward transmission: A) individual qPCR positivity and symptoms of malaria in household members; B) individual qPCR positivity and self-reported malaria; and C) individual qPCR positivity and qPCR positivity in household members.

The results of the sensitivity analysis were consistent with the main results, although, as expected, there were fewer episodes of parasitemia. When restricting the definition of qPCR positivity to those with parasite levels of at least 10 parasites/μL, only 66 episodes of parasitemia were identified in 57 individuals. Full results of the sensitivity analyses are shown in the supplementary materials.

## Discussion

In a two-year longitudinal cohort study in a low transmission setting in southern Zambia, the prevalence of parasitemia was low but persistent throughout the study period and across a wide age range. Parasite density was quite low and most episodes of parasitemia were transient, subsequently either cleared by the immune system or below the limit of detection. There was no evidence to support the hypothesis that low-density parasitemia led to symptomatic malaria. Despite the fact that asymptomatic parasitemia clustered in households within the same month, consistent with the idea of shared household risk factors and consistent with prior research demonstrating spatial clustering of asymptomatic parisitemia. there was no association with an increased risk of parasitemia or reported clinical malaria among household members in subsequent months that would be consistent with onward transmission (13,17). These findings suggest that, in this low transmission setting in southern Zambia, programmatic efforts to identify and treat low level parasitemia of this magnitude are not needed to prevent clinical malaria or achieve malaria elimination.

Asymptomatic parasitemia was not associated with typical malaria risk factors of sex, travel, or net usage. In low transmission settings, older individuals are expected to have premunition and thus higher odds of asymptomatic parasitemia of lower density, whereas younger children are assumed to be relatively immunologically naive and to have symptomatic disease when parasitemia (18). Instead, there were no significant decreases in parasite density by age and asymptomatic parasitemia was prevalent in children younger than five years.

Other studies have shown that any level of parasitemia carries a risk of onward transmission (19,20). Currently, Southern Province, Zambia deploys a reactive test-and-treat strategy following focal investigations, in which a malaria case at a health center triggers a reactive response around the case’s residence. RDTs are used for screening, which would have missed nearly all the parasitemic individuals identifiedin this cohort. To detect these low-level parasitemias, more sensitive tools are needed (21), but even newer ultra-sensitive RDTs would not have detected the level of parasitemia identified in this study (22).

Inherent in interview-based surveys are biases that may come with self-reporting. For example, net usage may be somewhat overestimated due to social desirability bias. However, this is an unavoidable limitation in this context, and the seasonal trends observed in net usage are unlikely to be differentially impacted. A more significant limitation is that most clinical cases were self-reported by individuals who sought care at a health facility not included in the study. However, because these individuals were followed monthly in this cohort, their recall for malaria diagnosis is expected to be relatively accurate. Under the assumption that these self-reported cases are wholly misclassified, the null finding that there is no relationship between clinical and asymptomatic infection may be incorrect, although the absence of clinical malaria in cohort members during the study period supports this conclusion – that these low level parasitemias have no clinical significance. Overall, the repeated longitudinal measurements in this cohort study minimize recall bias and the design is an overall strength.

Low-level parasitemia was prevalent in this low transmission setting in southern Zambia. There was no evidence that low-level asymptomatic parasitemia led to clinical malaria or transmission to other household members. Thus, programmatic efforts to identify and treat individuals with low level parasitemia of this magnitude is not warranted to minimize the disease burden or achieve malaria elimination.

## Supplementary Material

Supplementary Files

This is a list of supplementary files associated with this preprint. Click to download.

- Supplement.LowdensityparasitemiaandclinicalrelevancecohortJan142026.docx

## Figures and Tables

**Table 1:** Longitudinal cohort member demographic characteristics by qPCR positivity for *Plasmodium falciparum*.

	Overall	Never positive*	Ever positive*	p-value
**Household**	n = 167	n = 80	n = 87	
Household size (median [IQR])	5.0 [3. 0, 7.0]	3.0 [2.0, 6.0]	6.0 [4.0, 8.0]	< 0.001
Household has at least one ITN (n, %)	131 (76.6)	54 (74.0)	68 (81.9)	0.314
IRS conducted in household during study period (n, %)	4 (2.0)	3 (3.8)	1 (1.1)	0.554
Household has more than one sleeping structure (n, %)	49 (28.7)	17 (23.3)	31 (37.3)	0.085
**Individuals**	n = 1071	n = 927	n = 144	
Number of follow-up visits (median [IQR])	9.0 [2.0, 16.0]	8.0 [2.0, 15.0]	15.5 [10.0, 19.0]	< 0.001
Age (years) (median [IQR])	16.0 [8.0, 28.0]	16.0 [9.0, 27.0]	13.0 [7.0, 28.3]	0.154
Female sex (n, % of column total)	133 (57.6)	122 (59.2)	11 (44.0)	0.215
Visitors (n, %)	149 (14.9)	142 (16.4)	7 (5.2)	0.001
Years of education (median [IQR])^b^	7.0 [1.0, 10.0]	7.0 [2.0, 10.0]	7.0 [0.0, 9.0]	0.185
Ever employed (n, %)^a,b^	369 (34.5)	311 (33.5)	58 (40.3)	0.137
Ever travelled (n, %)^c^	572 (53.4)	484 (52.2)	88 (61.1)	0.057
% nights using bed net (mean (SD))	34% (37%)	34% (38%)	38% (35%)	0.246

* Samples were considered qPCR positive if at least one of the two wells had a parasite density above one parasite/μL and a melting point within ± 0.5° Celsius of the control melting point. Never positive individuals were qPCR negative at every study visit. Ever positive individuals were qPCR positive at one or more study visits.

Abbreviations: IQR (inter quartile range);

a p-values for variables reported as % (n) are from Fisher’s exact test and variables reported as mean or median, (IQR) are from the Kruskal-Wallis test;

b Among adults, 16 years of age and older;

c Among residents and temporary residents

## Data Availability

The data used to support the findings of this study are not publicly available. However, investigators interested in the data can submit a written request to the corresponding author Dr. William Moss (wmoss1@jhu.edu) and the Macha Research Trust IRB Chairperson (mrt.irb@macharesearch.org, +260979402560) to assess the request.
